# The Effectiveness and Safety of Serious Games for Improving Cognitive Abilities Among Elderly People With Cognitive Impairment: Systematic Review and Meta-Analysis

**DOI:** 10.2196/34592

**Published:** 2022-03-10

**Authors:** Alaa Abd-alrazaq, Mohannad Alajlani, Dari Alhuwail, Carla T Toro, Anna Giannicchi, Arfan Ahmed, Ahmed Makhlouf, Mowafa Househ

**Affiliations:** 1 Division of Information and Computing Technology College of Science and Engineering Hamad Bin Khalifa University, Qatar Foundation Doha Qatar; 2 AI Center for Precision Health Weill Cornell Medicine-Qatar Doha Qatar; 3 Warwick Manufacturing Group Institute of Digital Healthcare University of Warwick Warwick United Kingdom; 4 Information Science Department College of Life Sciences Kuwait University Kuwait Kuwait; 5 Health Informatics Unit Dasman Diabetes Institute Kuwait Kuwait; 6 Behavioral Health Services and Policy Research Department New York State Psychiatric Institute New York, NY United States; 7 Ambulance Service Hamad Medical Corporation Doha Qatar

**Keywords:** serious games, cognitive training, exergames, mild cognitive impairment, Alzheimer disease, dementia, global cognition, systematic review, meta-analysis

## Abstract

**Background:**

Cognitive impairment is a mental disorder that commonly affects elderly people. Serious games, which are games that have a purpose other than entertainment, have been used as a nonpharmacological intervention for improving cognitive abilities. The effectiveness and safety of serious games for improving cognitive abilities have been investigated by several systematic reviews; however, they are limited by design and methodological weaknesses.

**Objective:**

This study aims to assess the effectiveness and safety of serious games for improving cognitive abilities among elderly people with cognitive impairment.

**Methods:**

A systematic review of randomized controlled trials (RCTs) was conducted. The following 8 electronic databases were searched: MEDLINE, Embase, CINAHL, PsycINFO, ACM Digital Library, IEEE Xplore, Scopus, and Google Scholar. We also screened reference lists of the included studies and relevant reviews, as well as checked studies citing our included studies. Two reviewers independently carried out the study selection, data extraction, risk of bias assessment, and quality of evidence appraisal. We used a narrative and statistical approach, as appropriate, to synthesize the results of the included studies.

**Results:**

Fifteen studies met the eligibility criteria among 466 citations retrieved. Of those, 14 RCTs were eventually included in the meta-analysis. We found that, regardless of their type, serious games were more effective than no intervention (*P*=.04) and conventional exercises (*P*=.002) for improving global cognition among elderly people with cognitive impairment. Further, a subgroup analysis showed that cognitive training games were more effective than no intervention (*P*=.05) and conventional exercises (*P*<.001) for improving global cognition among elderly people with cognitive impairment. Another subgroup analysis demonstrated that exergames (a category of serious games that includes physical exercises) are as effective as no intervention and conventional exercises (*P*=.38) for improving global cognition among elderly people with cognitive impairment. Although some studies found adverse events from using serious games, the number of adverse events (ie, falls and exacerbations of pre-existing arthritis symptoms) was comparable between the serious game and control groups.

**Conclusions:**

Serious games and specifically cognitive training games have the potential to improve global cognition among elderly people with cognitive impairment. However, our findings remain inconclusive because the quality of evidence in all meta-analyses was very low, mainly due to the risk of bias raised in the majority of the included studies, high heterogeneity of the evidence, and imprecision of total effect sizes. Therefore, psychologists, psychiatrists, and patients should consider offering serious games as a complement and not a substitute to existing interventions until further more robust evidence is available. Further studies are needed to assess the effect of exergames, the safety of serious games, and their long-term effects.

## Introduction

### Background

Societies globally are rapidly aging at unprecedented rates. The United Nations projects that by 2050, 1 in 6 people in the world will be over the age of 65 years [1]. The aging population requires special care and attention due to the emergence of several progressive complex health issues, including hearing loss, vision impairments, physical ailments, noncommunicable diseases (diabetes), and mental and cognitive disorders [2]. With the growing size of the geriatric population, the World Health Organization has recommended the prevention of cognitive decline to be ranked as a global mental health priority [3].

Unfortunately, declining mental and cognitive abilities not only affect people and their relatives but also burden health care systems. Among the top chronic diseases causing the progressive decline and deterioration of cognitive abilities are mild cognitive impairment (MCI), Alzheimer disease (AD), and dementia. Globally, it is estimated that the number of prevalent dementia cases more than doubled (117%) between 1990 and 2016 [4]. Moreover, globally, it has been estimated that there are about 40 million people aged over 65 years who have dementia, and AD affects about 70% of them [5]. Therefore, preventing and managing age-related cognitive abilities and functions are important public health issues.

Research suggests that cognitive symptoms experienced by people with declining or deteriorating cognitive abilities are often associated or even preceded by behavioral symptoms. Therefore, treatments for improving cognitive functions and abilities often cannot be separated from behavioral treatments [6], and the treatments include pharmacological and nonpharmacological interventions. If implemented effectively, nonpharmacological interventions, such as lifestyle, good nutrition, exercise, and serious games, can delay the onset of dementia and cognitive decline [7-10]. Serious games are defined as games that have a purpose other than entertainment, such as education, prevention, screening, diagnosing, and therapeutic rehabilitation [11-13]. With the ubiquity and accessibility of technology and handheld devices, these serious games have been integrated into personal computers, game consoles (eg, Xbox), and, more recently, smartphones and tablets [3]. This review focused on digital serious games that are used as a therapeutic rehabilitation. Serious games exist in a variety of formats based on the therapeutic modality, including (1) cognitive training games that aim to maintain or improve users’ cognitive functions, such as working memory and attention; (2) exergames, or video games that entail physical exercises (eg, balance exercises) as part of the intended gameplay; (3) computerized cognitive behavioral therapy (CBT) games, which are video games that provide CBT for the users; and (4) biofeedback games, which are video games that utilize electrical sensors attached to the participant to receive information about the participant’s body state (eg, electrocardiogram sensors) and seek to influence some of the player body’s functions (eg, heart rate). Previous systematic reviews have shown that serious games have the potential to prevent or alleviate mental disorders such as depression [14] and anxiety [15]. Further, the literature suggests that serious games can be a good mental stimulant and improve brain health through the use of memory, visualization, and motor skills [16].

### Research Gap and Aim

Numerous prior studies have investigated the effectiveness of serious games for improving cognitive abilities. Aggregating and summarizing the findings from these studies is crucial for drawing conclusions about the effectiveness of serious games for improving cognitive abilities. Several systematic reviews have aggregated evidence from these studies; however, they are undermined by certain shortcomings that limit the generalization of the findings. Specifically, these reviews (1) focused on older adults who did not have cognitive impairment [3,17,18]; (2) included pilot randomized controlled trials (RCTs) and/or quasiexperiments [18-20]; (3) conducted the search a long time ago (5 years ago) [18,19]; (4) did not assess the quality of evidence [3,18-20]; (5) did not assess the safety of serious games [17-20]; or (6) only focused on specific types of serious games, such as cognitive training games [3,19] and exergames [18,20]. To address the existing gaps in the literature, this review aims to assess the effectiveness and safety of serious games for improving cognitive abilities among elderly people with cognitive impairment.

## Methods

### Overview

We conducted a systematic review and meta-analysis following the PRISMA (Preferred Reporting Items for Systematic Reviews and Meta-Analyses) guidelines (Multimedia Appendix 1) [21]. The protocol for this review is registered at PROSPERO (ID: CRD42021272757).

### Search Strategy

#### Search Sources

The following 8 bibliographic databases were searched in order to retrieve studies that were relevant to this review: MEDLINE (via Ovid), PsycInfo (via Ovid), Embase (via Ovid), CINAHL (via EBSCO), IEEE Xplore, ACM Digital Library, Scopus, and Google Scholar. The search was conducted on August 6, 2021, by the first author (A Abd-alrazaq). In order to retrieve studies that were added to the databases after that date, an automatic alert was set up, and it ran its course for 12 weeks (ending on November 5, 2021). Due to the large number of studies retrieved on Google Scholar, only the first 10 pages (ie, 100 hits) were considered, as they are automatically ordered based on their relevance [22]. We applied backward reference list checking, which involves the screening of reference lists of the included studies and relevant reviews. Further, the studies that cited the included studies were screened (forward reference list checking).

#### Search Terms

For developing the search query for this review, we consulted 2 experts in digital mental health and checked the search query used in other systematic reviews within this field. The chosen search terms related to the target population (eg, cognitive impairment), target intervention (eg, serious games and exergames), and target study design (eg, RCT and clinical trial). Multimedia Appendix 2 summarizes the search query that was used for searching each of the 8 databases.

### Study Eligibility Criteria

Only RCTs that assessed the effectiveness of serious games for improving cognitive abilities among elderly people with cognitive impairment were included in this study. For the purpose of this review, the target intervention was serious games that are available on digital platforms, such as computers, consoles (Xbox, PlayStation, etc), mobile phones, tablets, handheld devices, Nintendo, and other computerized devices. Further, gaming had to be an integral and primary component of the intervention and used solely for the purpose of therapy. Studies combining serious games with other interventions were eligible if the control group received the same adjacent intervention. Nondigital games and those used for other purposes, such as monitoring, screening, diagnosis, and research, were excluded.

The population of interest was elderly people (≥60 years) with cognitive impairment/disorder (eg, MCI, AD, or dementia). Their diagnosis had to be confirmed by examining the inclusion criteria or baseline scores against standardized diagnostic criteria (eg, Mini-Mental State Examination [MMSE] and Montreal Cognitive Assessment [MoCA]). Studies about healthy elderly people without cognitive impairment, health care providers, and caregivers were excluded. No restrictions were applied regarding gender and ethnicity.

The main outcome of interest in this review was global cognition regardless of the tool used for measuring the outcome. Global cognition is a general measure of all cognitive abilities such as memory, language, learning, and attention. Although behavioral outcomes relate to cognitive outcomes, behavioral outcomes are out of the scope of this review. The secondary outcome of interest was adverse events, which we used as an indicator of the safety of serious games. Studies were excluded if they assessed only cost effectiveness, acceptance, feasibility, and satisfaction. This review focused on outcome data that were measured immediately after the intervention rather than follow-up data.

All types of RCTs (parallel, cluster, crossover, or factorial) were included, but pilot or feasibility RCTs, quasiexperiments, observational studies, and reviews were excluded. For practical reasons, only those trials in the English language were eligible for inclusion. Studies published from 2010 onwards were included. Studies published as journal articles, conference proceedings, and dissertations were included. Otherwise, conference abstracts and posters, preprints, commentaries, proposals, and editorials were all excluded. No restrictions related to the country of publication, comparator, and study settings were applied.

### Study Selection

Relevant studies were identified in the following steps. To begin, the obtained studies were imported into EndNote to identify and delete duplicate items. Following the PRISMA guidelines, the titles and abstracts of all retrieved studies were evaluated in the second phase by 2 reviewers working independently. Two reviewers independently evaluated the entire text of the studies included in the previous step. Any disagreements were resolved via discussion. The 2 reviewers were the first 2 authors of this paper: A Abd-alrazaq and MA. The interrater agreement (Cohen κ) in steps 2 and 3 were 0.84 and 0.89, respectively, indicating a near-perfect level of interrater agreement [23].

### Data Extraction

Data from the included papers were extracted by 2 reviewers (A Abd-alrazaq and MA) independently using Microsoft Excel. Multimedia Appendix 3 outlines the data extraction form used to extract data from the included studies. Furthermore, we pilot tested the form with 2 of the included studies. Disagreements among the reviewers (A Abd-alrazaq and MA) were settled via discussion. An interrater agreement of 0.88 was observed, indicating a near-perfect degree of agreement. Contact was made with the first and corresponding authors in an attempt to retrieve metrics, such as mean, standard deviation, and sample size, if they were unavailable from the published studies.

### Risk of Bias Appraisal

Cochrane Collaboration recommends assessment of the risk of bias by 2 independent reviewers using the Risk-of-Bias 2 (RoB-2) tool [24], and as such, these guidelines were followed for this review. The RoB-2 tool appraises the risk of bias in the following 5 domains in RCTs: randomization process, deviations from intended interventions, missing outcome data, measurement of the outcome, and selection of the reported result [24]. The risk of bias judgments in these domains are used to determine the overall risk of bias for each included study. Any inconsistencies in decisions between the reviewers were solved via consulting a third reviewer. There was near-perfect interrater agreement between the reviewers (Cohen κ=0.81) [23].

### Data Synthesis

For the purpose of synthesizing the gathered data, a narrative and statistical approach was employed. Texts and tables were used to describe the features of the included studies (demographic, intervention, comparator, and outcome measures) in our narrative synthesis. The results of the experiments were compiled and categorized based on the comparator as follows: control, conventional exercises, conventional cognitive training, and other serious games. A meta-analysis was conducted when at least two studies of the same comparator submitted enough data (ie, mean, standard deviation, and number of participants in each intervention group). Meta-analyses were conducted using Review Manager (RevMan 5.4). The standardized mean difference (SMD) (Cohen *d*) was used to assess the overall effect of each study, as the type of data for the outcome of interest (global cognition) was continuous and the instruments used to evaluate the outcome were diverse among the included trials. Due to the high clinical heterogeneity between the meta-analyzed studies in terms of serious game characteristics (eg, its types, duration, frequency, and period), population characteristics (eg, sample size, mean age, and health condition), and outcome measures (ie, tools and follow-up period), the random effects model was used for the analysis.

If we observed a statistically significant difference between groups when a meta-analysis was conducted, we further sought to examine if it was clinically significant. The term “minimal clinically important difference” (MCID) refers to the smallest change in a measured outcome that a patient would consider worthwhile and significant enough to merit a change in treatment. The MCID bounds were computed as ±0.5 times the meta-analyzed studies’ SMD.

In order to examine the degree and statistical significance of heterogeneity in the meta-analyzed studies, we calculated I^2^ and the chi-square *P* value, respectively. A chi-square *P* value of .05 or less suggests heterogeneous meta-analyzed studies [25]. When I^2^ ranged from 0% to 40%, 30% to 60%, 50% to 90%, and 75% to 100%, the degree of heterogeneity was judged as insignificant, moderate, substantial, and considerable, respectively [25].

In order to assess the overall quality of evidence resulting from the meta-analyses, we used the Grading of Recommendations Assessment, Development, and Evaluation (GRADE) approach [26]. The GRADE approach appraises the quality of evidence based on the following 5 domains: risk of bias, inconsistency (ie, heterogeneity), indirectness, imprecision, and publication bias [26]. Two reviewers assessed the overall quality of the meta-analyzed evidence, and differences in decisions were addressed by discussion. There was near-perfect interrater agreement among the reviewers (Cohen κ=0.92) [23].

## Results

### Search Results

**Figure 1:**
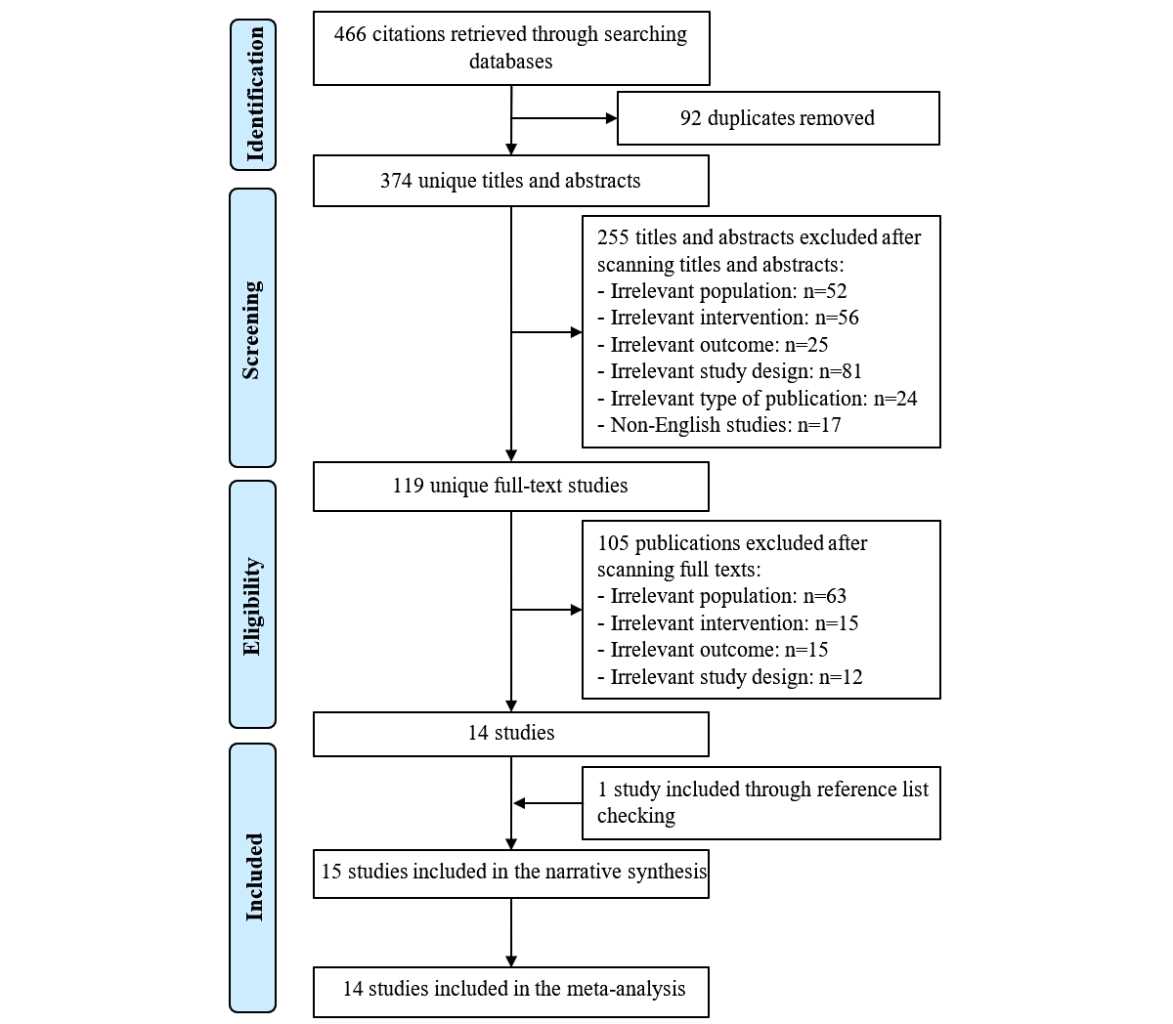
Flow chart of the study selection process.

By searching the 7 electronic databases, 466 records were retrieved (Figure 1). Of these records, 92 duplicates were identified using the software EndNote and were excluded. Checking titles and abstracts of the remaining articles led to the exclusion of 255 records for the following reasons: (1) participants were younger than 60 years and/or without cognitive impairment (n=52); (2) interventions were not serious games (n=56); (3) the outcome was not global cognition (n=25); (4) the study design was not RCT (n=81); (5) studies were not peer-reviewed articles, theses, or conference proceedings (n=24); and (6) the articles were published in languages other than English (n=17). Reading the full text of the remaining 119 publications led to the exclusion of 105 publications for the following reasons: (1) participants were younger than 60 years and/or without cognitive impairment (n=63); (2) interventions were not serious games (n=15); (3) the outcome was not global cognition (n=15); and (4) study design was not RCT (n=12). One additional study was found through backward reference list checking. In total, 15 RCTs were included in the current review [27-41]. All studies were included in meta-analyses, except 1 study [41].

### Characteristics of the Included Reviews

The included studies were published between 2013 and 2021 (Table 1). The year in which the largest number of included studies were published was 2020 (n=3). The included studies were conducted in 13 different countries, and there was a general equal distribution of studies in these countries. All included studies were peer-reviewed journal articles, except for 1 book chapter included. The trial type used in most included studies was parallel RCT (n=13).

The sample size in the included studies ranged from 20 to 114, with an average of 70.4. The mean age of participants reported in 14 studies ranged between 67 and 84.2 years, with an average of 75.2 years. The percentage of males reported in 14 studies ranged from 23.5% to 70%, with an average of 44.5%. The mean MMSE score for participants in the included studies varied between 10.2 and 27. Participants in the included studies had MCI (n=10), AD (n=2), dementia (n=1), MCI and dementia (n=1), and neurocognitive disorders (n=1). Participants were recruited from clinical settings in 13 studies and the community in 2 studies.

Serious games alone were used as interventions in 13 of the included studies, whereas the remaining 2 studies used serious games combined with other interventions (Table 2). The included studies used 19 different serious games. Some studies used more than one game. Serious games used in the included studies were grouped into the following 2 types based on the therapeutic modality that they delivered: cognitive training games (n=12) and exergames (n=3). Games were designed with a “serious” purpose from the beginning (designed serious games) in 14 studies. However, in the remaining study, games were not designed as serious games from the start but rather were used for a serious purpose (purpose-shifted games). The most common platforms used for playing the games were computers (n=8). Serious games were played under the supervision of health care providers or caregivers in 9 studies, but were not played under any supervision in 3 studies. In the remaining studies, the serious games were both supervised as well as unsupervised. The duration of the games in the included studies ranged between 25 and 100 minutes, with a 30-minute duration in one-third of the included studies (n=5). The frequency of playing the games varied between 2 times a week and 5 times a week, but it was 2 times a week in about half of the studies (n=8). The period of interventions ranged from 4 weeks to 25 weeks, but was less than 13 weeks in two-thirds of the studies (n=10).

The comparison groups received passive interventions in 9 studies, whereas active interventions were received in 8 studies (eg, conventional exercises and conventional cognitive activities) (Table 3). Two studies delivered both active and passive interventions as comparators. The duration of the active comparators ranged between 25 and 100 minutes. The frequency of the active comparators varied between 1 time a week and 7 times a week. The period of the active comparators varied between 6 weeks and 25 weeks. The outcome of interest (ie, global cognition) was measured using 7 different tools, but the most common tool used by the included studies was the MMSE (n=11). The outcome of interest was measured immediately after the intervention in all included studies, and the longest follow-up period was 24 weeks. Participant attrition was reported in 14 studies and ranged from 0 to 28.

**Table 1:** Characteristics of the studies and population.

First author	Year	Country	Publication type	RCT^a^ type	Sample size, n	Mean age (years)	Sex (male), %	MMSE^b^ score	Health condition	Setting
Savulich [27]	2017	United Kingdom	Journal article	Parallel	42	76.1	59.5%	26.7	MCI^c^	Clinical
Yang [28]	2017	South Korea	Journal article	Parallel	20	71	70%	23.1	AD^d^	Clinical
Tarnanas [29]	2014	Greece	Book chapter	Parallel	114	70.3	39%	26.4	MCI	Clinical
Cavallo [30]	2016	Italy	Journal article	Parallel	80	76.4	36.3%	22.9	AD	Clinical
Zhuang [31]	2013	China	Journal article	Parallel	33	83.1	24.2%	10.2	MCI, dementia	Clinical
Thapa [32]	2020	South Korea	Journal article	Parallel	68	72.7	23.5%	26.2	MCI	Clinical
Jahouh [33]	2021	Spain	Journal article	Parallel	80	84.2	44%	22.2	MCI	Clinical
Robert [34]	2020	France	Journal article	Parallel	46	79.4	47.8%	21.4	Cognitive disorders	Clinical
Singh [35]	2014	Australia	Journal article	Factorial	100	70.1	32%	27	MCI	Community
van Santen [36]	2020	Netherlands	Journal article	Cluster	112	79	53.5%	18.6	Dementia	Clinical
Liao [37]	2021	Taiwan	Journal article	Parallel	61	81.5	32.6%	22.9	MCI	Community
Amjad [38]	2019	Pakistan	Journal article	Parallel	44	NR^e^	NR	24	MCI	Clinical
Hagovská [39]	2015	Slovakia	Journal article	Parallel	80	67	51.2%	26	MCI	Clinical
Hagovská [40]	2016	Slovakia	Journal article	Parallel	80	67	51.2%	26.4	MCI	Clinical
Gooding [41]	2015	United States	Journal article	Parallel	96	75.6	58.1%	NR	MCI	Clinical

^a^RCT: randomized controlled trial.

^b^MMSE: Mini-Mental State Examination.

^c^MCI: mild cognitive disorder.

^d^AD: Alzheimer disease.

^e^NR: not reported.

**Table 2:** Characteristics of the interventions.

First author	Intervention	Serious game name	Serious game type	Platform	Supervision	Duration (min)	Frequency (times/week)	Period (weeks)
Savulich [27]	Serious games	Game Show	Cognitive training game	Tablet	Supervised	60	2	4
Yang [28]	Serious games	Brain-Care	Cognitive training game	PC	Unsupervised	60	2	12
Tarnanas [29]	Serious games	Virtual Reality Museum	Cognitive training game	VR^a^ headset	Supervised	90	2	21
Cavallo [30]	Serious games	Brainer	Cognitive training game	PC	Supervised	30	3	12
Zhuang [31]	Serious games	NR^b^	Cognitive training game	PC	Supervised	75	3	24
Thapa [32]	Serious games	Juice making, Crow Shooting, Love house, Fireworks	Cognitive training game	VR headset, hand controllers	Supervised	100	3	8
Jahouh [33]	Serious games	Step, Nodding	Exergame	Wii console	Supervised	40-45	2-3	8
Robert [34]	Serious games	MeMo	Cognitive training game	PC, tablet	Unsupervised	30	4	12
Singh [35]	Serious games	COGPACK	Cognitive training game	PC	Supervised	75	2	25
van Santen [36]	Serious games	NR	Exergame	Stationary bike, screen	Unsupervised	NR	5	25
Liao [37]	Serious games	Tano and LongGood	Exergame	Kinect, VR headset	Supervised	60	3	12
Amjad [38]	Serious games	Body and Brain Exercises	Cognitive training game	Xbox console, Kinect	Supervised	25-30	5	6
Hagovská [39]	Serious games + conventional exercises	CogniPlus	Cognitive training game	PC	Both	30	2	10
Hagovská [40]	Serious games + conventional exercises	CogniPlus	Cognitive training game	PC	Both	30	2	10
Gooding [41]	Serious game	BrainFitness	Cognitive training game	PC	Both	60	2	17

^a^VR: virtual reality.

^b^NR: not reported.

**Table 3:** Characteristics of comparators and outcomes.

First author	Comparator	Duration (min)	Frequency (times/week)	Period (weeks)	Outcome measures	Follow-up	Attrition, n
Savulich [27]	Control	N/A^a^	N/A	N/A	MMSE^b^	Postintervention	0
Yang [28]	Control	N/A	N/A	N/A	MMSE, CDR^c^	Postintervention	0
Tarnanas [29]	Control, conventional cognitive activities	90	2	21	MMSE	Postintervention	9
Cavallo [30]	Control	30	3	12	MMSE	Postintervention, 24-week follow-up	4
Zhuang [31]	Control	N/A	N/A	N/A	ACE-R^d^	Postintervention	10
Thapa [32]	Control	30-50	1	8	MMSE	Postintervention	2
Jahouh [33]	Control	N/A	N/A	N/A	MMSE, GDS^e^	Postintervention	NR^f^
Robert [34]	Control	N/A	N/A	N/A	MMSE	Postintervention, 12-week follow-up	3
Singh [35]	1: Conventional exercises + sham cognitive training 2: Serious games + conventional exercises 3: Control	1: 75 2: 100 3: 60	2	25	ADAS-Cog^g^	Postintervention, 74-week follow-up	14
van Santen [36]	Conventional exercises	N/A	5	25	MMSE	Mid-intervention, Postintervention	28
Liao [37]	Conventional exercises	60	3	12	MoCA^h^	Postintervention	15
Amjad [38]	Conventional exercises	25-30	5	6	MMSE, MoCA	Postintervention	6
Hagovská [39]	Conventional exercises	30	7	10	MMSE	Postintervention	2
Hagovská [40]	Conventional exercises	30	7	10	ACE	Postintervention	2
Gooding [41]	Empirically validated serious game, commercially available serious game	60	2	17	MMSE + WAIS-R^i^	Postintervention	22

^a^N/A: not applicable.

^b^MMSE: Mini-Mental State Examination.

^c^CDR: Clinical Dementia Rating Scale.

^d^ACE-R: Addenbrooke Cognitive Examination-Revised.

^e^GDS: Global Deterioration Scale.

^f^NR: not reported.

^g^ADAS-Cog: Alzheimer Disease Assessment Scale-Cognitive.

^h^MoCA: Montreal Cognitive Assessment.

^i^WAIS-R: Wechsler Adult Intelligence Scale-Revised.

### Results of Risk of Bias Appraisal

**Figure 2:**
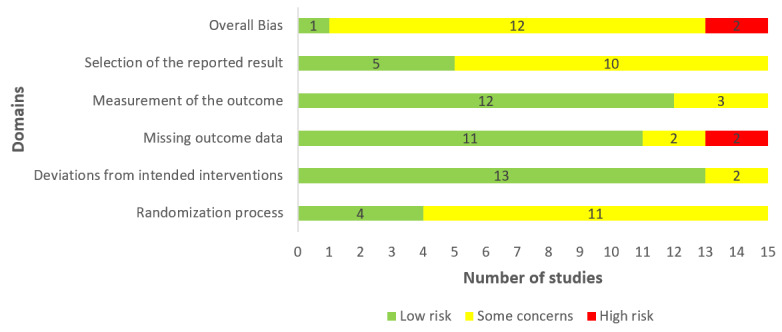
Review authors’ judgements about each “risk of bias” domain.

Seven included studies generated an appropriate random allocation sequence for the randomization process. In 4 studies, the allocation sequence was concealed until participants were assigned to interventions. There were no imbalances between groups at baseline in 14 studies. Consequently, the risk of bias due to the randomization process was rated as low in only 4 out of 15 studies (Figure 2).

Participants and those who delivered the interventions were aware of the assigned interventions during the trial in 13 and 14 studies, respectively. In all included studies, there was no evidence that the experimental contexts led to a deviation from the intended intervention. All 15 studies used appropriate analysis methods (eg, intention-to-treat analysis) to estimate the effect of the intervention. According to these judgments, the risk of bias due to the deviations from the intended interventions was low in 13 out of 15 studies (Figure 2).

Missing outcome data were less than 5% in 7 studies. In only 1 study, there was evidence that the findings were not biased by missing outcome data. The missing outcome data could be related to participants’ health status in 3 studies. Consequently, 11 studies were judged as having a low risk of bias in the “missing outcome data” domain (Figure 2).

In all included studies, global cognition was assessed using appropriate measures, and measurement methods were comparable across intervention groups. The assessor of the outcome was blinded to the assigned interventions in 9 studies. Assessment of the outcome may have been affected by knowledge of the intervention received in 3 studies. Accordingly, the risk of bias in the “measuring the outcome” domain was rated as low in 13 studies (Figure 2).

Five studies published their protocol in sufficient detail. In all studies, reported outcome measurements did not differ from those specified in the analysis plan, and there was no evidence that studies selected their results from the many results produced from multiple eligible analyses of the data. Based on these judgments, the risk of bias due to the selection of the reported results was considered low in 5 studies (Figure 2).

In the last domain “overall bias,” the risk of bias was considered high in 2 studies as they were judged as having a high risk of bias in at least one domain. Twelve studies raised some concerns in the domain of overall bias as they had some issues in at least one of the domains and were not at high risk for any domain. The remaining study was judged to be at low risk of bias for the domain of overall bias given that it was rated to be at low risk of bias for all domains. Reviewers’ judgments about each “risk of bias” domain for each included study are presented in Multimedia Appendix 4.

### Results of Studies

In this review, serious games were compared with control (no/passive interventions), conventional exercises, conventional cognitive activities, and other serious games. Results of the included studies are shown in the following subsections based on these comparisons. Then, the results of the subgroup analysis are shown. Lastly, the results of the included studies regarding the safety of serious games have been reported.

#### Serious Games Versus No/Passive Interventions

The effect of serious games was compared with a control (no/passive interventions) in 9 studies [27-35]. Passive interventions refer to interventions that do not have a known effect on the measured outcome, such as reading newspapers articles, surfing the internet, and watching a documentary program. While 6 studies did not find a statistically significant difference in global cognition between the groups [30-35], the 3 remaining studies showed a statistically significant difference in global cognition between the groups, favoring serious games over no intervention [27-29].

**Figure 3:**
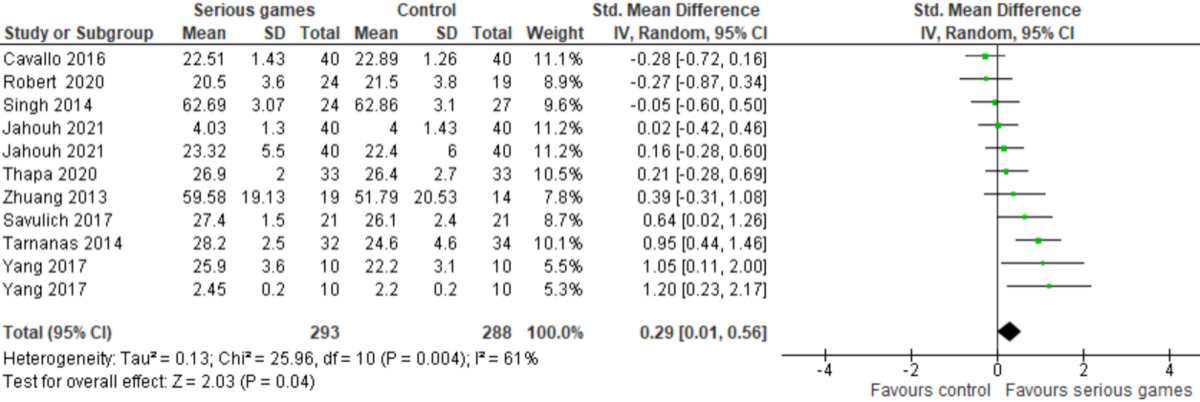
Forest plot of 9 studies (11 comparisons) comparing the effect of serious games to a control in terms of global cognition.

The results of these 9 studies were included in the meta-analysis. Two of these studies assessed global cognition using 2 different measures (MMSE and Clinical Dementia Rating Scale [28], and MMSE and Global Deterioration Scale [GDS] [33]). Therefore, we included the results of all these measures in the meta-analysis to form 11 comparisons (Figure 3). The meta-analysis showed a statistically significant difference in global cognition (*P*=.04) between the serious games and control groups, favoring serious games over no/passive intervention (SMD 0.29, 95% CI 0.01-0.56). This difference was also clinically important as the overall effect was outside MCID boundaries (−0.15 to 0.15) and its CI did not cross the “no effect” line (zero effect). For this outcome, MCID boundaries were calculated as ±0.5 times the SMD value (0.29). The statistical heterogeneity of the evidence was substantial (*P*=.004, I^2^=61%). The quality of the evidence was very low as it was downgraded by 5 levels due to a high risk of bias, heterogeneity, and imprecision (Multimedia Appendix 5).

#### Serious Games Versus Conventional Exercises

The effect of serious games was compared with conventional exercises in 6 studies [35-40]. While 3 studies did not find a statistically significant difference in global cognition between the groups [35-37], the 3 remaining studies showed a statistically significant difference in global cognition between the groups, favoring serious games over conventional exercises [38-40].

**Figure 4:**
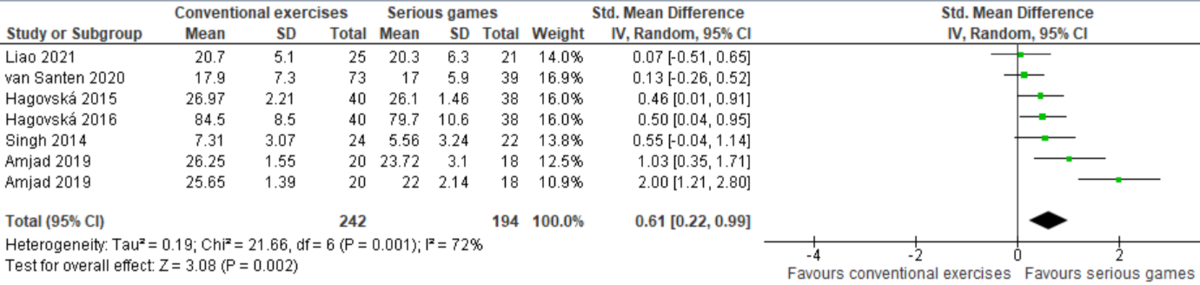
Forest plot of 5 studies (7 comparisons) comparing the effect of serious games to conventional exercises in terms of global cognition.

The results of these 6 studies were included in the meta-analysis. One of these studies assessed global cognition using the following 2 different measures: MMSE and MoCA [38]. Therefore, we included the results of the 2 measures in the meta-analysis to form 7 comparisons (Figure 4). The meta-analysis showed a statistically significant difference in global cognition (*P*=.002) between the groups, favoring serious games over conventional exercises (SMD 0.61, 95% CI 0.22-0.99). This difference was also clinically important as the overall effect was outside MCID boundaries (−0.31 to 0.31) and its CI did not cross the “no effect” line (zero effect). For this outcome, MCID boundaries were calculated as ±0.5 times the SMD value (0.61). The statistical heterogeneity of the evidence was substantial (*P*=.001, I^2^=72%). Like the meta-analysis seen in the previous section, the quality of this evidence was very low as it was downgraded by 5 levels due to a high risk of bias, heterogeneity, and imprecision (Multimedia Appendix 5).

#### Serious Games Versus Other Interventions

One study compared the effect of serious games to conventional cognitive activities in terms of global cognition among patients with MCI [29]. The study found no statistically significant difference between the groups in global cognition as measured by the MMSE (*P*=.05) and GDS (*P*=.07) [29].

Gooding et al [41] compared the effect of a serious game (serious game 1 that involves computerized cognitive training [BrainFitness]) to another serious game (serious game 2 that incorporates empirically validated motivational teaching and rehabilitation techniques into computerized cognitive training) and various commercially available computer games and puzzles (ie, BrainAge, Sudoku, and crossword puzzles) [41]. The study found a statistically significant difference in global cognition between the groups, favoring serious game 1 and serious game 2 over commercially available computer games and puzzles. However, there was no significant difference in global cognition between the serious game 1 group and serious game 2 group [41].

#### Subgroup Analysis

**Figure 5:**
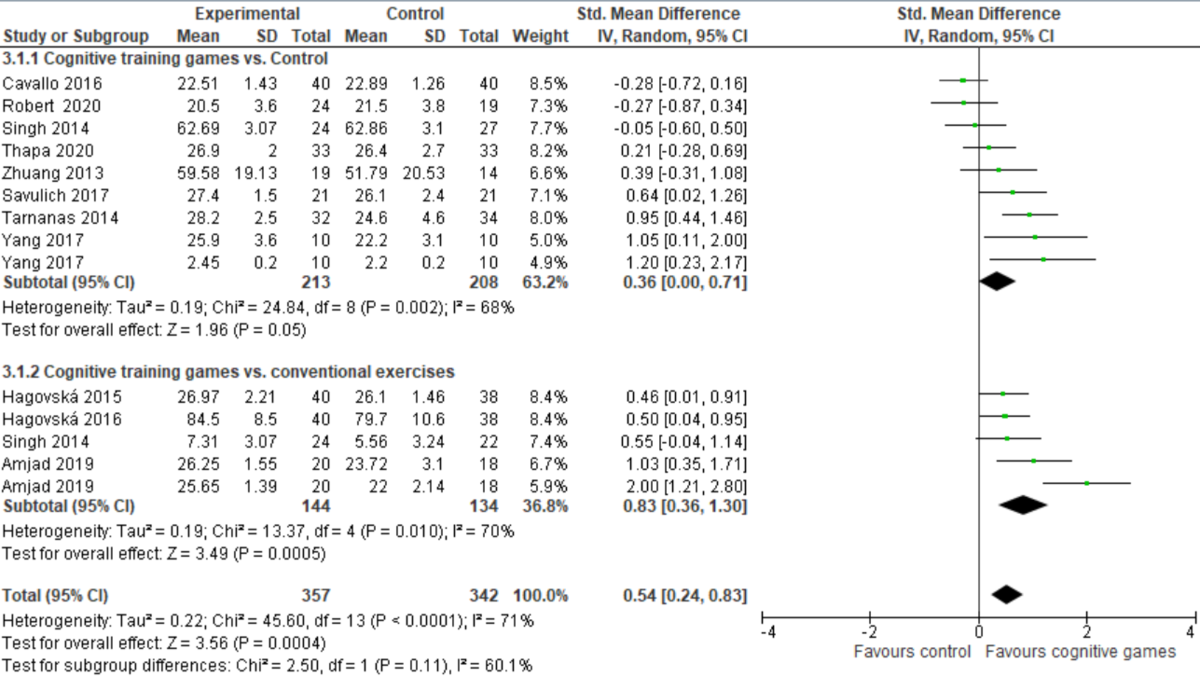
Forest plot of 10 studies (14 comparisons) comparing the effect of cognitive training games to control and conventional exercises in terms of global cognition.

We conducted subgroup analyses to investigate whether different types of serious games (ie, cognitive training games and exergames) have a different effect on global cognition. A subgroup analysis of 11 studies (14 comparisons) showed that cognitive training games had a statistically significant effect on global cognition compared to control (*P*=.05) and conventional exercises (*P*<.001) (Figure 5). The overall effect of cognitive training games on global cognition was statistically significant (*P*<.001) compared with both control and conventional exercises (SMD 0.54, 95% CI 0.24-0.83). This difference was also clinically important as the overall effect was outside MCID boundaries (−0.27 to 0.27) and its CI did not cross the “no effect” line (zero effect). For this outcome, MCID boundaries were calculated as ±0.5 times the SMD value (0.54). The statistical heterogeneity of the evidence was substantial (*P*<.001, I^2^=71%). The quality of this evidence was very low as it was downgraded by 5 levels due to a high risk of bias, heterogeneity, and imprecision (Multimedia Appendix 5).

**Figure 6:**
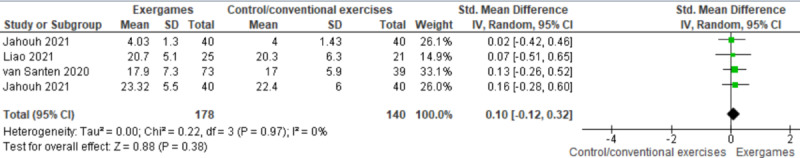
Forest plot of 3 studies (4 comparisons) comparing the effect of exergames to control and conventional exercises in terms of global cognition.

A subgroup analysis of 3 studies (4 comparisons) showed no statistically significant difference (*P*=.38) in global cognition between the exergame group and control or conventional exercise group (SMD 0.10, 95% CI −0.12 to 0.32) (Figure 6). The statistical heterogeneity of the evidence was a concern in this analysis (*P*=.97, I^2^=0%). The quality of the evidence was very low as it was downgraded by 4 levels due to a high risk of bias and imprecision (Multimedia Appendix 5).

#### Safety of Serious Games

Five studies assessed the safety of serious games by checking adverse events [34-38]. Of these, 2 studies did not find any adverse events of the interventions during the study period [37,38]. The 3 remaining studies spotted some adverse events, but they were comparable between groups [34-36]. Specifically, Fiatarone Singh et al [35] found 6 adverse events distributed as follows: 3 events in the serious game plus conventional exercises group, 1 event in the conventional exercises group, 0 events in the serious game group, and 2 events in the control group. The adverse events reported in this study were falls during assessment (n=3) and exacerbations of pre-existing arthritis symptoms (n=3). In the second study [36], the mean number of falls was not statistically different between the exergame group and control group after the intervention (1.2 vs 2.0) and after a 6-month follow-up (1.5 vs 1.5). The last study reported that there was 1 adverse event during the study period, which occurred in a participant from the serious game group [34].

## Discussion

### Principal Findings

This review investigated the effectiveness of serious games for improving global cognition as reported by RCTs. Very low–quality evidence from 9 RCTs (11 comparisons) and 6 RCTs (7 comparisons) showed that the effect of serious games on global cognition was statistically significant in comparison with no/passive interventions and conventional exercises. None of the previous reviews examined the effect of all types of serious games. Due to evidence paucity, no statistical analysis was carried out to compare serious games to other types of interventions (other serious games and conventional cognitive activities).

Additionally, very low–quality evidence from 11 RCTs (14 comparisons) showed that the effect of cognitive training games on global cognition was statistically significant in comparison with no/passive interventions, conventional exercises, and both. Interestingly, the effect of serious games in comparison with conventional exercises was higher than their effect in comparison with no/passive interventions. Studies in both meta-analyses were comparable in terms of population, intervention, and outcome measures. This paradoxical finding may be attributed to the fact that passive interventions used in the included studies (reading newspapers articles, playing games, solving puzzles, surfing the internet, and participating in educational programs on general health care) improved global cognition among participants, and thereby, the difference in global cognition between the serious game group and passive intervention group decreased. This is evident in Figure 3, where studies that used passive interventions showed either a negative effect size (which means that passive interventions had a higher effect than serious games) [30,35] or a small positive effect size (which indicates that passive interventions and serious games have a comparable effect) [32] in comparison with studies that used no interventions.

Our findings are in line with the findings of a previous review that compared the effect of cognitive training games to passive interventions, active interventions, and both in terms of global cognition among old adults with MCI [19]. Specifically, a meta-analysis of 10 RCTs (12 comparisons) in that review showed a statistically significant difference in global cognition between the groups, favoring cognitive training games over passive interventions, active interventions, and both [19]. It is worth mentioning that the review by Hill et al [19] is different from the current review in several aspects: (1) the review by Hill et al focused only on a specific type of serious games, which is cognitive training games; (2) it included pilot RCTs and/or quasiexperiments; (3) it did not assess the quality of evidence; (4) it conducted the search a long time ago (5 years ago); and (5) it did not assess the safety of serious games.

Very low–quality evidence from 3 RCTs (4 comparisons) showed an insignificant effect of exergames on global cognition in comparison with both control and conventional exercises. Our findings are inconsistent with findings from a previous review that compared the effect of exergames to both active and passive interventions in terms of global cognition among adults with and without health issues [18]. Specifically, a meta-analysis of 17 RCTs in that review showed a statistically significant difference (*P*<.001) in global cognition between the groups, favoring exergames over both active and passive interventions [18]. This inconsistency in findings can be attributed to several factors: (1) participants had cognitive impairment in only 2 of the 17 studies in that review [18]; (2) the mean age of participants were less than 60 years in 3 studies [18]; (3) the review included pilot RCTs and/or quasiexperiments; (4) the review included a large number of studies in the meta-analysis in comparison to the current review; and (5) the quality of evidence in the current review was very low.

According to 5 of the included studies, there was no significant difference in the number of adverse events between groups, indicating that serious games are safe. This finding was also concluded by a previous review about the use of cognitive training games for improving cognitive abilities among elderly people without cognitive impairment [3].

### Strengths and Limitations

#### Strengths

In comparison with previous reviews [3,17-20], the current review is the only one that assessed the effectiveness of both serious games and their types. Given that this review strictly adhered to highly recommended guidelines for reporting systematic reviews (ie, PRISMA), it can be deemed a transparent and high-quality review. We included only RCTs as it is the most rigorous research method for studying cause-effect relationships [42]; therefore, this review’s conclusion is likely more credible.

There is no concern about the risk of publication bias as the authors sought to identify as many relevant studies as possible by searching the most popular databases in the information technology and health fields, searching grey literature databases, conducting backward and forward reference list checking, and using a well-developed search query.

Given that all processes (ie, study selection, data extraction, risk of bias assessment, and quality of evidence evaluation) were carried out by 2 reviewers independently, the risk of selection bias is not a concern in this review. This review enables the reader to draw more accurate conclusions given that we appraised the quality of the evidence using the GRADE approach. When possible, we synthesized data statistically, and this improved the power of studies and increased the estimates of the likely size of the effect of serious games on global cognition.

#### Limitations

The effectiveness and safety of serious games delivered on nondigital platforms and those used for other purposes (eg, screening or diagnosis) cannot be commented on, because this review excluded studies discussing these types of serious games. This review focused on the effectiveness and safety of serious games for promoting global cognition among elderly people with cognitive impairment; thus, the effectiveness and safety of serious games for improving specific cognitive abilities (eg, memory, learning, and executive functions) or behavioral outcomes (eg, apathy, depression, and agitation) among other age groups without cognitive impairment cannot be commented on.

We excluded numerous studies as they were quasiexperiments, pilot RCTs, published before 2010, or written in non-English languages. Therefore, it is likely that we missed some relevant studies. We excluded these studies as quasiexperiments and pilot RCTs have lower internal validity than RCTs [42]. Because of practical constraints, it was not possible to translate all non-English studies. We included studies published from 2010 onwards given that previous reviews found a few studies published before 2010, and serious games have greatly advanced in the last decade.

This review focused on the short-term effect of serious games by meta-analyzing only postintervention data rather than follow-up data, because only 3 studies reported follow-up data and the follow-up period was not consistent between studies. Therefore, we cannot comment on the long-term effect of serious games on global cognition. The quality of the evidence in all meta-analyses was very low, and this may decrease the internal validity of our findings.

This review used postintervention data for each group to assess the effect size for each meta-analyzed study rather than the pre-post intervention change for each group, and thereby, it is likely that the effect size was overestimated or underestimated in this review. We used postintervention outcome data because the majority of studies did not report the mean and standard deviation for the pre-post intervention change in global cognition for each group, and there was no statistically significant difference in global cognition at baseline between groups in all studies.

Non-RCTs can be used to assess the safety of interventions, such as serious games. However, such studies were not included in this review. Thus, it is likely that we missed several studies about the safety of serious games.

### Research and Practical Implications

#### Research Implications

Given that this review focused on the effectiveness and safety of serious games for improving global cognition among elderly people with cognitive impairment, it is recommended that researchers conduct further reviews to assess the effectiveness and safety of serious games for improving specific cognitive abilities (eg, executive function, processing speed, memory, and learning) among people from different age groups with or without cognitive impairment.

A few studies were carried out in developing countries, and as such, the generalizability of this review’s findings to such countries may be limited. More studies should be conducted in developing countries, especially given the varying nature of their cultures and socioeconomic conditions. A handful of studies assessed the safety of using serious games for improving cognitive abilities; thus, more studies will be helpful to draw more definitive conclusions about the safety of serious games.

This review did not assess the long-term effect of serious games given the lack of studies that reported follow-up data. Researchers should follow-up with participants to assess the long-term effect of serious games on global cognition. The majority of the included studies did not report the mean and standard deviation for the pre-post intervention change in global cognition for each group. It is important that future studies report this information to calculate more accurate effect sizes.

The overall risk of bias was low in only 1 study given that most studies had issues mainly in the randomization process and selection of the reported results. Accordingly, researchers should follow recommended guidelines or tools (eg, RoB-2 [24]) when conducting and reporting RCTs to avoid the above-mentioned biases. Although many studies examined the effect of exergames on global cognition among healthy older people [18], only 3 studies in this review examined their effect among older people with cognitive impairment. We encourage researchers to bridge this gap by conducting more studies about the effect of exergames on global cognition among elderly people with cognitive impairment.

#### Practical Implications

This review showed that serious games and specifically cognitive training games are more effective than no intervention and conventional exercises for improving global cognition, whereas exergames are as effective as no intervention and conventional exercises. However, these findings should be interpreted carefully because the quality of evidence in all meta-analyses was very low given that the majority of the included studies were judged to have some concerns in overall bias, the heterogeneity of the evidence was high in all meta-analyses except 1, and the total effect sizes were imprecise. Accordingly, psychologists and psychiatrists should consider offering serious games as a complement and not as a substitute to existing interventions until more robust evidence is available.

Still, the emerging evidence from this study presents promising opportunities to leverage serious games to alleviate the burden on health care systems due to exponential growth in the number of elderly people worldwide in the years to come. Serious games can allow elderly people with cognitive impairments to improve their psychological, physiological, sensory/motor, and social functions, thereby enjoying a higher quality of living [12]. Because many elderly people live in isolation and experience a lack of social interactions, which in turn can contribute to mortality and morbidity [43], serious games can promote social bonding with family and friends by being played in a comfortable environment (ie, homes) [44].

Mobile devices (smartphones and tablets) were used as the platform for serious games in only 2 studies. Mobile devices are particularly appealing as they are cheaper than computers and more pervasive than gaming consoles. Mobile devices are also more accessible than computers and gaming consoles. It is estimated that there were about 15 billion mobile devices and more than 7.1 billion mobile users worldwide in 2021 [45]. App and game developers should collaborate to develop serious games that target cognitive abilities and can be played via mobile devices.

When examining the few studies conducted in developing countries, it seems that there is more focus on implementing serious games in developed countries despite the greater shortage of mental health professionals in developing countries (1 per 10,000,000 people [46]). Therefore, more serious games should be developed in developing countries to improve cognitive abilities among elderly people with cognitive impairment.

### Conclusion

Serious games and specifically cognitive training games have the potential to improve global cognition among elderly people with cognitive impairment. However, definitive conclusions could not be drawn regarding the effectiveness and safety of serious games for improving global cognition among elderly people with cognitive impairment. This is because the quality of evidence in all meta-analyses was very low mainly due to concerns raised about the bias in the majority of the included studies, high heterogeneity of the evidence, and imprecision of total effect sizes. Therefore, psychologists, psychiatrists, and patients should consider serious games as a complement and not as a substitute to existing interventions until further more robust evidence is available. Further reviews are required to assess the effectiveness and safety of serious games for improving specific cognitive abilities (eg, executive function, processing speed, memory, and learning) among people from different age groups with or without cognitive impairment. Additional studies are needed to assess the effect of exergames, the safety of serious games, and their long-term effects.

## Appendix

Multimedia Appendix 1 PRISMA (Preferred Reporting Items for Systematic Reviews and Meta-Analyses) checklist.

Multimedia Appendix 2 Search strategy.

Multimedia Appendix 3 Data extraction form.

Multimedia Appendix 4 Reviewers’ judgements about each “risk of bias” domain for each included study.

Multimedia Appendix 5 GRADE (Grading of Recommendations Assessment, Development, and Evaluation) profile for comparison of serious games to control or conventional exercises for global cognition.

## Abbreviations

AD: Alzheimer disease
CBT: cognitive behavioral therapy
GDS: Global Deterioration Scale
GRADE: Grading of Recommendations Assessment, Development, and Evaluation
MCI: mild cognitive impairment
MCID: minimal clinically important difference
MMSE: Mini-Mental State Examination
MoCA: Montreal Cognitive Assessment
PRISMA: Preferred Reporting Items for Systematic Reviews and Meta-Analyses
RCT: randomized controlled trial
RoB-2: Risk-of-Bias 2
SMD: standardized mean difference
