# Supplementary material for: The Effectiveness and Safety of Serious Games for Improving Cognitive Abilities Among Elderly People With Cognitive Impairment: Systematic Review and Meta-Analysis
Source: JMIR Serious Games. 2022 Mar 10;10(1):e34592. doi: 10.2196/34592 (PMC8949701; doi:10.2196/34592)
Supplement: Multimedia Appendix 4 [file games_v10i1e34592_app4.docx]

**Appendix 4 Reviewers’ judgements about each “risk of bias” domain for each included study**
