# Supplementary material for: The Effectiveness and Safety of Serious Games for Improving Cognitive Abilities Among Elderly People With Cognitive Impairment: Systematic Review and Meta-Analysis
Source: JMIR Serious Games. 2022 Mar 10;10(1):e34592. doi: 10.2196/34592 (PMC8949701; doi:10.2196/34592)
Supplement: Multimedia Appendix 5 [file games_v10i1e34592_app5.docx]

| **Appendix 5 GRADE (Grading of Recommendations Assessment, Development, and Evaluation) profile for comparison of serious games to control or conventional exercises for global cognition** | | | | | | | | | | | |
| --- | --- | --- | --- | --- | --- | --- | --- | --- | --- | --- | --- |
| **Certainty assessment** | | | | | | | **Summary of findings** | | | | |
| **Participants (studies) Follow-up** | **Risk of bias** | **Inconsistency** | **Indirectness** | **Imprecision** | **Publication bias** | **Overall certainty of evidence** | **Study event rates (%)** | | **Relative effect (95% CI)** | **Anticipated absolute effects** | |
|  |  |  |  |  |  |  | **With Control** | **With Serious games** |  | **Risk with Control** | **Risk difference with Serious games** |
| **Serious games compared to control for global cognition** | | | | | | | | | | | |
| 581 (9 RCTs)  (11 comparisons) | very serious^a^ | very serious^b^ | not serious | serious^c,d^ | none | ⨁◯◯◯ Very low | 288 | 293 | - | - | SMD **0.29 higher** (0.01 higher to 0.56 higher) |

| **Serious games compared to conventional exercises for global cognition** | | | | | | | | | | | | | | | | | | | | |
| --- | --- | --- | --- | --- | --- | --- | --- | --- | --- | --- | --- | --- | --- | --- | --- | --- | --- | --- | --- | --- |
| 436 (6 RCTs)  (7 comparisons) | very serious^e^ | very serious^f^ | | not serious | | serious^c,g^ | | none | | ⨁◯◯◯ Very low | | 194 | | 242 | | - | | - | | SMD **0.61 higher** (0.22 higher to 0.99 higher) |
| **Cognitive training games compared to Control & Conventional exercises for Global cognition** | | | | | | | | | | | | | | | | | | | | |
| 699 (11 RCTs)  (14 comparisons) | very serious^h^ | very serious^i^ | not serious | | serious^c,j^ | | none | | ⨁◯◯◯ Very low | | 342 | | 357 | | - | | - | | SMD **0.54 higher** (0.24 higher to 0.83 higher) | |

| **Exergames compared to Control & Conventional exercises for Global cognition** | | | | | | | | | | | |
| --- | --- | --- | --- | --- | --- | --- | --- | --- | --- | --- | --- |
| 318 (3 RCTs) | very serious^k^ | not serious | not serious | very serious^l,m^ | none | ⨁◯◯◯ Very low | 140 | 178 | - | - | SMD **0.1 higher** (0.12 lower to 0.32 higher) |

**CI:** confidence interval; **SMD:** standardised mean difference

#### Explanations

a. Evidence was downgraded by 2 levels because there were some concerns in 9 studies due to issues mainly in the randomization process and selection of the reported results.

b. Evidence was downgraded by 2 levels as P=0.004 and I square=61%, indicating high heterogeneity.

c. Evidence was downgraded by 1 level because 95% CI crosses one of MID boundaries for this outcome.

d. MID for this outcome, calculated as ± 0.5 times the standardized mean difference (SMD), is ± 0.15

e. Evidence was downgraded by 2 levels because there were some concerns in 5 studies due to issues mainly in the randomization process and selection of the reported results.

f. Evidence was downgraded by 2 levels as P=0.001 and I square=72%, indicating high heterogeneity.

g. MID for this outcome, calculated as ± 0.5 times the standardized mean difference (SMD), is ± 0.31

h. Evidence was downgraded by 2 levels because there were some concerns in 9 studies due to issues mainly in the randomization process and selection of the reported results.

i. Evidence was downgraded by 2 levels as P=0.001 and I square=71%, indicating high heterogeneity.

j. MID for this outcome, calculated as ± 0.5 times the standardized mean difference (SMD), is ± 0.27.

k. Evidence was downgraded by 2 levels because there were some concerns in 3 studies due to issues mainly in the randomization process and missing outcome data.

l. Evidence was downgraded by 2 levels because 95% CI crosses the two MID boundaries for this outcome.

m. MID for this outcome, calculated as ± 0.5 times the standardized mean difference (SMD), is ± 0.05
